# Targeting the Platelet-Activating Factor Receptor (PAF-R): Antithrombotic and Anti-Atherosclerotic Nutrients

**DOI:** 10.3390/nu14204414

**Published:** 2022-10-20

**Authors:** Rajendran Harishkumar, Sakshi Hans, Janelle E. Stanton, Andreas M. Grabrucker, Ronan Lordan, Ioannis Zabetakis

**Affiliations:** 1 Department of Biological Sciences, University of Limerick, V94 T9PX Limerick, Ireland; 2 Bernal Institute, University of Limerick, V94 T9PX Limerick, Ireland; 3 Health Research Institute, University of Limerick, V94 T9PX Limerick, Ireland; 4 Cellular Neurobiology and Neuro-Nanotechnology Laboratory, Department of Biological Sciences, University of Limerick, V94 T9PX Limerick, Ireland; 5 Institute for Translational Medicine and Therapeutics, Perelman School of Medicine, University of Pennsylvania, Philadelphia, PA 19104, USA; 6 Department of Medicine, Perelman School of Medicine, University of Pennsylvania, Philadelphia, PA 19104, USA; 7 Department of Systems Pharmacology and Therapeutics, Perelman School of Medicine, University of Pennsylvania, Philadelphia, PA 19104, USA

**Keywords:** platelet-activating factor, platelet-activating factor receptor, polar lipids, antithrombotic activity, inflammation, atherosclerosis

## Abstract

Platelet-activating factor (PAF) is a lipid mediator that interacts with its receptor (PAF-R) to carry out cell signalling. However, under certain conditions the binding of PAF to PAF-R leads to the activation of pro-inflammatory and prothrombotic pathways that have been implicated in the onset and development of atherosclerotic cardiovascular diseases (CVD) and inflammatory diseases. Over the past four decades, research has focused on the identification and development of PAF-R antagonists that target these inflammatory diseases. Research has also shown that dietary factors such as polar lipids, polyphenols, and other nutrient constituents may affect PAF metabolism and PAF-R function through various mechanisms. In this review we focus on the inhibition of PAF-R and how this may contribute to reducing cardiovascular disease risk. We conclude that further development of PAF-R inhibitors and human studies are required to investigate how modulation of the PAF-R may prevent the development of atherosclerotic cardiovascular disease and may lead to the development of novel therapeutics.

## 1. Introduction

Atherosclerotic cardiovascular diseases (CVD) are the leading cause of morbidity and mortality globally [1]. Various factors contribute to the development of atherosclerosis, but evidence in recent decades has demonstrated that nutrition plays a pivotal role in the prevention of atherosclerosis and other chronic inflammatory conditions including diabetes and obesity [2,3]. Hence there is a requirement to research the effects of diets and food components on cardiovascular health. 

Atherosclerosis is a progressive inflammatory disease responsible for the development of atherothrombotic complications including myocardial infarction, peripheral artery disease, and ischaemic or transient stroke among other cardiac manifestations [4,5]. Atherosclerosis develops through several steps including endothelial dysfunction followed by the deposition of lipids in the intima, which accumulate in the lining of blood vessels. These lipids are then engulfed by macrophages, which eventually undergo apoptosis forming foam cells and a necrotic core that leads to the development of the characteristic lesions or fatty streaks in blood vessels. Erosion of these lesions or plaques causes microruptures that activate platelets causing fibrin netting and platelet aggregates to form on the inner walls of arteries, thus leading to the narrowing of blood vessels affecting blood supply [6]. With time, the lumen may narrow and erode further causing plaque rupture, leading to a major cardiovascular event such as myocardial infarction or stroke. The main mechanistic events that lead to these events are characterised by persistent low-grade inflammation [5]. 

However, inflammation is a necessary physiological response of the innate immune system, and its main role is to maintain a constant internal environment despite being subjected to constantly changing environmental pressures. These can include mechanical, physical, chemical, infectious, immunological, or reactive natural adverse events. The inflammatory response seeks to diminish and/or minimize the agents that causes tissue damage, promote adequate wound healing, and restore tissue homeostasis. However, if the inflammatory response fails to resolve owing to the persistence of the triggering factors or poor restoration of the original tissue, a prolonged underlying inflammatory process arises, leading to increased tissue dysfunction and adverse effects. At the molecular and cellular level, it has been postulated that endothelial dysfunction leading to systemic inflammation appears to be the primary underlying mechanistic factor in the onset and progression of atherosclerosis [7]. Endothelial dysfunction is often defined by an inflammatory microenvironment that acts on leukocytes and endothelial cells via interactions with other immune cells such as T lymphocytes, mast cells, dendritic cells (DC), and platelets [8].

Platelets play a key role in the onset and development of atherosclerosis [9,10,11,12,13]. Platelets also orchestrate the development of obstructive thrombi in the latter stages of the atherosclerotic process in response to plaque rupture through the sequential processes of haemostatic responses to vascular injury such as initiation, extension, and stabilization [14]. Each of these stages contains pro-haemostatic molecular mechanisms, in balance with anti-haemostatic processes, which restrict the reaction to the damage site and prevent inappropriate vascular occlusion. The molecular players involved in the initiation process include adhesion molecules, signalling ligands, and their associated platelet surface receptors [15]. Strong inflammatory and prothrombotic mediators such as platelet-activating factor (PAF) play pivotal roles in these processes, particularly in the activation of platelets [16]. Indeed, PAF and its receptor have previously been investigated as a pharmaceutical target for some inflammatory conditions including asthma and sepsis with limited success to date. They have also been implicated in many of the key processes that lead to the development of atherosclerosis. However, researchers over the years have postulated that dietary PAF-R antagonists may affect PAF-related signalling and inflammatory pathways [7,17,18]. This has opened several avenues of research that aim to investigate certain dietary patterns such as the Mediterranean diet, which is thought to offer protection from atherosclerotic cardiovascular disease and other inflammatory diseases due to a high concentration of these compounds in the diet [18,19]. In this review, we examine the role of various nutrients and their effects on PAF and its receptor PAF-R and how attenuating this inflammatory and thrombotic pathway may contribute to atherosclerosis prevention via altering one’s diet. It is also important to recognise that while this review largely focusses on the relationship between PAF and the PAF-R, there are also ongoing developments in cardiovascular research relating to the metabolic enzymes of PAF, which have been discussed at length elsewhere [7,20].

## 2. Platelet-Activating Factor (PAF) and PAF-Receptor (PAF-R)

PAF (1*-O*-alkyl-2-acetyl-s*n*-glycero-3-phosphocholine) is a phospholipid mediator that functions through the PAF-receptor (PAF-R). PAF was discovered when Ig-E sensitised basophils of rabbits were challenged with antigen stimuli [21]. In physiology, PAF is an important signalling molecule in the renal, cardiovascular, immune, and reproductive systems. However, PAF is not just one molecule; there happens to be a family of PAF-like lipids (PAFLL) or PAF-like moieties, which all have varying degrees affinity with the PAF-R leading to various levels of potency [22]. The classic PAF molecule has an alkyl ether linkage at the *sn*-1 position, a characteristic acetyl group at the *sn*-2 position, and a phosphocholine group at the *sn*-3 position of the glycerol backbone [23]. The most potent PAF molecules contains a 16:0 at the *sn*-1 position, but may also have 18:0, 17:0, and 18:1 on the alkyl ether-linked side chain leading to varying degrees of affinity for the PAF-R and as a consequence, varying degrees of biological activity [7,24]. PAF is known to carry out its biological activities at concentrations as low as 10^−12^ M and almost always by 10^−9^ M as an intercellular messenger [25] and it carries out its functions in a autocrine, juxtracrine, and paracrine manner [26,27]. The history of the elucidation of the PAF structure and developments in the field has recently been reviewed [28]. 

**Figure 1:**
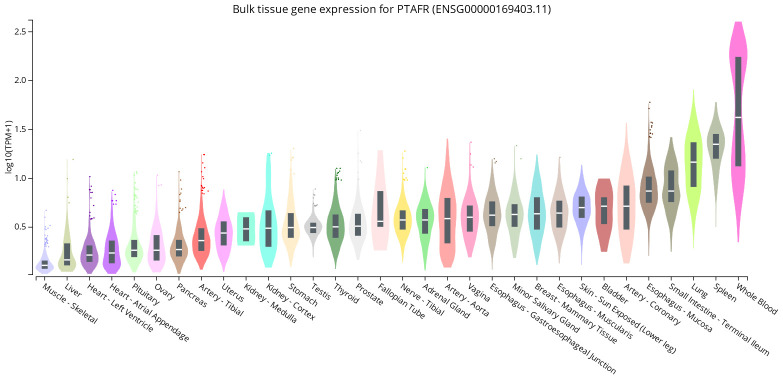
Bulk gene expression for the platelet-activating factor receptor (PAF-R) encoded by the gene *PTAFR* in various human tissues using data from the Genotype-Tissue Expression (GTEx) Project [30]. The expression data is shown in transcripts per million (TPM) with the plots showing the median and the 25th and 75th percentiles. Dots indicate outliers, which are above or below 1.5 times the interquartile range.

The PAF-R is expressed by cells in various tissues, including the lungs, spleen, heart, kidneys, skeletal muscle, and in blood cells as shown in Figure 1 [24]. Therefore, it is also unsurprising that PAF-R signaling is implicated in many physiological processes [28]. There is an abundance of phospholipids in the brain and central nervous system (CNS) [29], where the PAF-R is expressed by various parts of the CNS including the spinal cord, substantia niagra, hypothalamus, hippocampus, frontal cortex, nucleus accumbens, cortex, cerebellum, cerebellar hemisphere, basal ganglia, and the amygdala [30]. Notably, PAF is also synthesised by neuronal tissue and its signaling is associated with neurotrophic effects [31]. Indeed, permeability of the blood-brain barrier (BBB) increases via PAF-R dependent mechanisms, consequent to calcium (Ca^2+^) influx, increased nitric oxide levels, and alterations to proteins that regulate intercellular gaps in the BBB *in vivo* [32]. 

PAF-R signalling also plays a prominent role in reproductive biology, including ovulation, fertilisation, preimplantation, and parturition in women. In men, PAF is present in spermatozoa and is thought to be involved in sperm motility and in the induction of acrosome reactions [33,34,35,36,37,38]. PAF and PAF-R is also a known physiological mediator of healthy cardiovascular function via modulating inflammatory signaling, platelet function, and blood pressure [39,40,41]. As the name suggests, PAF is a platelet activator via binding to the PAF-R in the normal response to injury [13]. PAF-R binding by PAF induces platelet shape change and the release of platelet granules via stimulation of the phosphatidylinositol cycle and intracellular Ca^2+^ mobilization. Serotonin and platelet factor 4 are secreted, along with arachidonic acid and other bioactive lipids, including PAF, which mediate platelet aggregation [13,42,43].

While we are still learning about the roles of PAF and PAF-R in physiology, PAF is mostly known for its role as an inflammatory messenger that passes signals to cell types such as platelets, neutrophils, endothelial cells, macrophages, and lymphocytes [7]. PAF is involved in multiple communicable and non-communicable diseases through excessive binding with the PAF-R. Some studies have shown that PAF mediates metastasis in tumour cells. For example, PAF triggers human melanoma cells via stimulating the phosphorylation of cAMP-responsive element (CRE)-binding protein (CREB) and activating transcription factor-1(ATF-1). This signal transduction leads to the overexpression of major effectors involved in tumour growth, angiogenesis, and malignant progressions such as MMPs, STAT-3, and NF-*κ*B [44]. PAF also affects other pathological processes including increased vascular permeability, hypotension, ulcerogenesis, bronchoconstriction triggering airway hyperresponsiveness, and platelet degranulation. PAF has also been implicated in septic shock, asthma, ischemia/ reperfusion injury, pancreatitis, inflammatory bowel disease, and rhinitis [45]. PAF-R activation has also been reported to manifest in communicable diseases. For example, PAF-R activation causes increased thrombocytopenia, haemoconcentration, increased systemic levels of cytokines, and lethality in wild-type mice compared with PAF-R-silenced mice in a model of dengue fever [46]. PAF is implicated in other infectious diseases characterised by inflammation including human immunodeficiency virus (HIV) [47] and severe acute respiratory syndrome coronavirus 2 (SARS-CoV-2) infection [48,49]. Considering the vast pathological functions of PAF and its receptor, many investigations have focused on preventing PAF from binding to the PAF-R with the aim of reducing prothrombotic and proinflammatory signalling. 

Structurally, the PAF-R is a seven transmembrane G-protein coupled receptor encoded by the *PTAFR* gene. The gene locus has been identified in humans as chromosome 1p35-p34.5. Human and guinea pig PAF receptors are single polypeptides with 342 amino acids; rat and mouse PAF receptors omit one amino acid in the third extracellular loop. Despite various findings to the contrary, it is presently believed that a single receptor subtype mediates all PAF’s actions in humans and is generally located on the plasma membrane, endomembrane, nucleus, and nuclear envelope [18,50]. The gene, *PTAFR*, is tightly regulated by two distinct promotors that are involved in the transcriptional regulation, consequently there are two alternatively spliced transcripts that differ in their untranslated regions. The first, transcript 1, is widely expressed in tissues regulated by inflammation, predominantly in leukocytes, macrophages, eosinophils, and monocytes. The second, transcript 2, is found in organs such as the heart, kidney, lung, and spleen and its expression can be influenced by oestrogen, thyroid hormone T3, retinoic acid, transforming growth factor-β (TGF-β), tumour necrosis factor-α (TNF-α), interferon-γ and others. The second transcript is not thought to be expressed by hematopoietic cells or in the brain [24,51,52]. In a positive feedback manner, PAF may upregulate the expression of its own receptor via transcript 1 through NF-κB signalling [53]. There is also evidence that PAF-R transcription is dependent on activation of the Jak/STAT pathway [51]. The upregulation of PAF synthesis and its degradation is also tightly regulated and has been extensively reviewed [16]. However, there are many aspects of PAF-R (*Ptafr*) expression that have been underexplored including whether it exhibits circadian rhythmicity as some data indicates that it might, along with genes associated with PAF metabolism, including *Pla2g7* (Circadb: Circadian Expression Profiles Data Base. Available online: http://circadb.hogeneschlab.org (accessed 12 October 2022)). Considering the PAF-R is expressed in numerous cells and tissue types there is a lot to left to be explored regarding its function and modulation.

**Figure 2:**
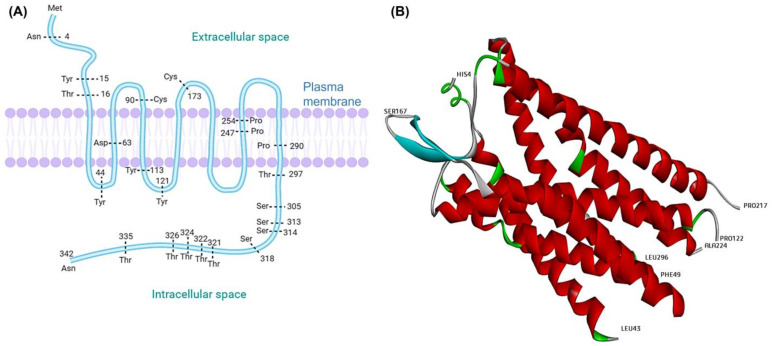
(**A**) Diagrammatic representation of the platelet-activating factor receptor (PAF-R) with its seven transmembrane domains within the plasma membrane bilayer [Note: PAF-R cloned from guinea pig represented with amino acid residues]; (**B**) Human PAF-R (Chain-A) with selective amino acid residues (PDB ID: 5ZKQ).

The first binding experiment of PAF was conducted on human platelets in 1982, whereby two distinct binding sites were revealed. The first site had shown higher affinity (K_d_ value = 37 ± 13 nm) and the other site possessed nearly low affinity toward PAF [54]. To understand the pathophysiological function of PAF, gene modifications were applied in earlier studies. For example, cDNA encoding the PAF-R was isolated from the guinea pig lung cDNA library and was cloned into *Xenopus laevis* oocytes depicted in Figure 2. In this cloned receptor, several amino acids are highly conserved when compared to other G protein receptors, including aspartic acid (Asp) in the second transmembrane segment, one cysteine (Cys) in both the second and third intracellular loops, and three proline (Pro) in the sixth and seventh segments. The PAF receptor’s cytoplasmic tail comprises four serines (Ser) and five threonines (Thr). There is a total of 12 tyrosine (Tyr) residues, with two of them located in the cytoplasmic loops. Asparagine (Asn) residues are found on the receptor’s exterior surface and may serve as sites for glycosylated residue attachment [55]. Some other reports stated that cloning of human PAF receptors can be achieved by isolating cDNA from peripheral leukocytes, heart, and EoL-1 eosinophilic leukaemia cells [56]. Figure 1B shows the helical 3D structure of PAF-R (Chain-A) that was obtained from the protein data bank (PDB ID: 5ZKQ) and its bound ligands were removed by UCSF Chimera [57].

### PAF and PAF-R Activation in Inflammatory Diseases

**Figure 3:**
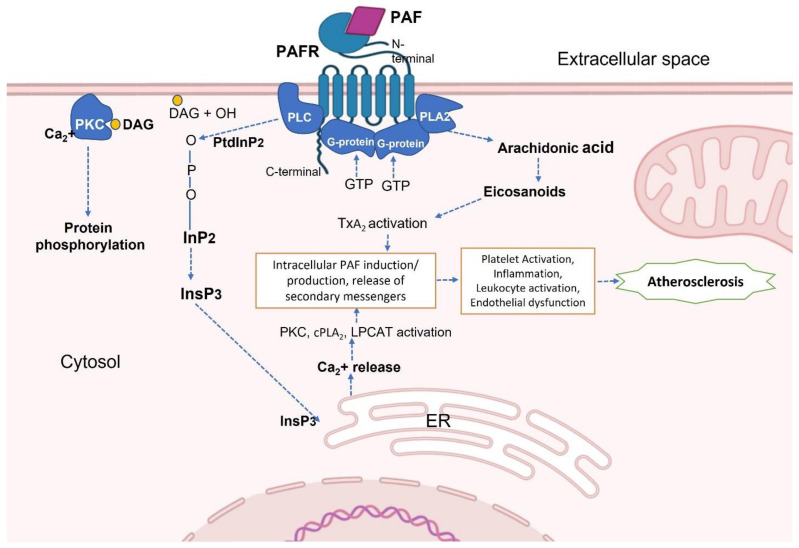
Mechanism of PAF-R activation and PAF-mediated signalling pathway [59,60]. Abbreviations: cPLA_2_—cytosolic phospholipase A_2_; DAG—Diacylglycerol; ER—Endoplasmic reticulum; InP_2_ —inositol 4,5-bisphosphate; InsP_3_—inositol 1,4,5-triphosphate; LPCAT—lysophosphatidylcholine acyltransferase; PKC—protein kinase C; PLA_2_—Phospholipase A_2_; PLC—Phospholipase C; PtdInP_2_—phosphatidyl 4,5-bisphosphate; TxA_2_—thromboxane A_2_.

Elevated levels of PAF can be detected in tissues affected by inflammatory diseases [7]. Excessive activation of the PAF-R via PAF and PAF-like lipids (PAF-LL) in inflammatory diseases induces several biological effects including systemic pro-inflammatory, prothrombotic, and pro-proliferative signalling. Indeed, delayed immune responses have also been reported and PAF-R signalling has been implicated in cancer development. Many malignant cells have been shown to overexpress PAF-R [58]. The PAF-R receptor is related to phosphoinositide metabolism via a G-protein that is also linked to phospholipases C and A_2_. PAF-R stimulation results in the brief synthesis of diacylglycerol, which activates protein kinase C, and inositol triphosphate, which triggers the release of internal calcium reserves [59]. The activation of PAF-R through PAF is represented the Figure 3.

PAF increases tyrosine phosphorylation of several proteins in neutrophils, macrophages, and platelets, as well as nuclear factor kappa B (NF-*k*B) activation and transcription of *c-fos* and c*-jun* in inflammatory cells. PAF can activate the mitogen-activated protein kinase (MAPK) kinase-3, a known activator of p38 MAPK, and the Jak/STAT pathway [59]. Following ligand activation, the PAF-R is degraded through both the proteasome and lysosomal pathways. 

While platelet activation leads to aggregation as part of normal haemostatic function, under acute or systemic inflammatory conditions PAF-R activation has been shown to induce various immune and inflammatory pathways [44] that can lead to both acute and chronic conditions. For example, PAF-R activation by PAF induces histamine and prostaglandin D_2_ release from mast cells [61,62] and it is involved in the chemotaxis of mast cells [63]. PAF has been shown to be a powerful chemoattractant for eosinophils [64,65] and it is responsible for the generation of chemokines and prostaglandins [65,66,67]. PAF along with leukotriene B4 (LTB4) and matrix metalloproteinase-9 (MMP-9) are involved in the accumulation of eosinophils in asthmatic airways via interleukin-8 (IL-8) stimulation of neutrophils [68]. Indeed, PAF has been shown to promote the recruitment of neutrophils and polymorphonuclear cells to inflammatory sites [7], and these cells can also generate PAF [7,69,70], which is thought to be one of the underlying mechanisms by which conditions such as atherosclerosis may propagate [7,71]. 

As a consequence of the wide-ranging inflammatory actions of PAF and the PAF-R, pharmaceutical companies and scientists have previously investigated the use of PAF inhibitors and developed pharmaceutical grade products to target these inflammatory pathways. These include products such as Lexipafant [72], Modipafant [73], and Rupatadine [74,75] among others that have previously reviewed [28] for the treatment of asthma, sepsis, and other conditions characterised by PAF-related inflammation. However, a recent study has shown that PAF and PAFLL can mediate nucleotide-binding domain, leucine-rich-repeat-containing protein 3 and never in mitosis A-related kinase 7 (NLRP3-NEK7) inflammasome induction in a PAF-R independent manner, which may explain observations of the ineffectiveness of many PAF-R antagonists [76] including those aforementioned. These findings may lead to further developments in our understanding of the role of PAF in diseases such as cancer and atherosclerosis considering the important role of the inflammasome in these diseases. Pharmaceuticals aside, research has also determined that there are a broad range of naturally occurring PAF-R antagonists present in certain edible plants and foods, which will be discussed in the ensuing sections. 

## 3. Antiplatelet Properties of Nutrients

Diet has long been associated with the maintenance of health and the prevention of disease. It is well established that healthy dietary patterns, such as the Mediterranean diet and the dietary approaches to stop hypertension (DASH) diet, may offer protection against the development of atherosclerosis and cardiovascular diseases [77,78]. With this knowledge, the functional foods, dietary supplements, and nutraceuticals industries have grown exponentially over the last two decades offering individuals food-derived and natural product derived constituents that may confer health benefits on the consumer [79]. Historically, many cultures turn to food and natural products as a source of healing in times of ill health. These practices are particularly prevalent in areas with indigenous rural communities. The World Health Organization (WHO) defines traditional medicine as “the total of knowledge, skills, and practices based on theories, beliefs, and experiences indigenous to different cultures, whether explicable or not, used in the maintenance of health as well as the prevention, diagnosis, improvement, or treatment of physical and mental illness” [80]. Some traditional medicine systems are supported by substantial literature and recordings of theoretical notions and practical abilities; others are passed down verbally from generation to generation. Until now the turn of the 20th century, the majority of the world’s population relied on their own traditional medicine to satisfy their primary health care requirements in several regions of the world [81]. Traditional medicine is commonly referred to as “complementary and alternative medicine” when practised outside of its traditional culture [82]. Traditional medicine is most popular and practiced nowadays in China, India, and many African nations among others [83].

In India, the traditional system of medicine (TSM) has been practiced before the adoption of modern medicine by traditional communities to heal any type of illness. These medical practices provide invaluable assistance in the healthcare system for current and future generations. The traditional systems of Indian medicine, presently known as the Indian System of Medicine (ISM), have a very solid conceptual foundation and have been practiced for a very long period. Ayurveda, Siddha, and Unani are three prominent traditional systems practiced in India [84]. Some Indian medicinal plants are reported to have antihyperlipidemic activity and anti-thrombolytic because of their antiplatelet aggregation activity and fibrinolytic activity [85]. Phytocompounds such as cudratricusxanthone A [86], withaferin A [87], and even some of the serine proteases were identified and tend to prevent clot formation [88]. 

Over the past three decades, there has been considerable research conducted investigating the potential antithrombotic and anti-inflammatory effects of dietary PAF inhibitors. In particular, there has been a focus on polar lipids, mainly phospholipids and sphingolipids, derived from natural sources such as plants and animal products, which exert antithrombotic activities due their inhibition of the PAF-R activation and other platelet agonists [28,89,90]. A recent study found that dietary supplementation with plant extract containing aloe gel, grape juice, green tea extract, etc. reduced platelet sensitivity upon stimulation with PAF [91]. In this study, it is not clear what constituent or combination of constituents are responsible for these observed effects. However, many compounds such as polyphenols, phenolipids, and polar lipids present in these capsule constituents have previously been associated with antiplatelet effects. For example, certain compounds isolated from *Spirulina* (blue-green algae) and other marine algae also possess bioactive properties beneficial to health, including antiplatelet, anti-inflammatory and antioxidant qualities. These qualities have been traced to the glycolipid sulphoquinovosyl diacylglycerol (SQDG) present in photosynthetic plants [92,93]. 

However, plants are not the only food-derived antithrombotic polar lipids. Fish-derived lipids also exhibit inhibitory properties against PAF. Polar lipid fractions isolated from cod (*Gadus morhua*) and salmon (*Salmo salar*) showed platelet inhibitory capabilities, suggesting that the consumption of such lipids could protect against cardiovascular disease [90,94]. Other animal foods, such as dairy products, notably yoghurt, also exhibit inhibitory activities against PAF *in vitro* [95,96]. In humans, intake of yoghurt enriched with polar lipids from olive oil by-products resulted in lower platelet sensitivity against PAF, and reduced low-grade inflammation, assessed by monitoring serum levels of IL-10 and IL-6 [97].

Many compounds derived from traditional herbal remedies also possess potent anti- PAF activity. Curcumin is a spice derived from turmeric and commonly used in Asian cuisines. A 1999 study found that curcumin inhibits platelet aggregation induced by agonists such as PAF, epinephrine, and ADP, via the inhibition of thromboxane production and Ca^2+^ signalling [98]. Another investigation found that extracts of several species of Malaysian medicinal plants exhibited significant inhibitory activity against PAF [99]. The Korean folk medicinal plant *Alpinia officinarum* is traditionally used to treat gastrointestinal diseases. Diarylheptanoid compounds were isolated from this plant and also showed a high inhibitory effect against platelet aggregation by PAF [45]. Apart from medicinal plants, plants that are commonly found in various diets also possess bioactive compounds with antithrombotic activities with various target mechanisms as listed in a Table 1.

## 4. Antiplatelet Properties of Polar Lipids

Polar lipids are amphipathic in nature, possessing both a hydrophilic head group and a hydrophobic tail. Polar lipids are key structural components of cellular membranes, and they play a role in signaling cascades with membrane proteins [128]. Polar lipids are mostly phospholipids and sphingolipids. In contrast, neutral lipids are non-polar and hydrophobic. Neutral lipids include triacylglycerols, cholesterols, waxes, fatty acids, and esters [129]. Polar lipids have been identified as PAF inhibitors that interact and inhibit the PAF-R through various mechanisms, both direct and indirect, as previously reviewed [7]. In contrast, neutral lipids mostly do not exhibit potent antiplatelet activities [130]. In the following sections we discuss the existing evidence involving *in vitro*, *in vivo*, and *ex vivo* studies that investigate the potential anti-PAF properties of polar lipids.

### 4.1. In Vitro Studies of Platelet-Activating Factor Receptor (PAF-R) Antagonists

Several *in vitro* studies have been published that reported that polar lipids exhibit antiplatelet properties likely mediated by interactions between the PAF-R. These polar lipids tend to be mostly researched in foods of animal origin, particularly dairy and marine sources. In dairy, it has been reported that the beneficial properties of polar lipids may be altered or enhanced by fermentation of the dairy product. Fermented dairy products, such as yoghurt and cheeses have also been noted for their high inhibitory activity against PAF and other agonists. Many fermented foods that are traditionally part of the Mediterranean diet are rich in omega-3 polyunsaturated fatty acids that support cardiovascular health [131]. Cheeses made from goat’s or sheep’s milk are an important part of the Greek diet. For example, the traditional Greek cheeses Kefalotyri and Ladotyri have strong inhibitory activity against PAF-induced platelet aggregation [96]. Certain bacterial cultures, such as *Lactobacillus acidophilus* and *Streptococcus thermophilus* can increase the bioactivity of ovine yoghurt milk and alters its anti-thrombotic activity in presence of PAF [132]. These starter cultures are capable of producing and altering bioactive polar lipids by some mechanism, possibly by producing antimicrobial peptides known as bacteriocins which can alter the fatty acid composition. The bacterium *L. acidophilus* has been shown to reduce PAF-induced inflammatory response in human intestinal cells [133]. A similar investigation [134] found that fermentation increases the antithrombotic properties of bovine dairy and plant-based dairy alternative drinks. Homemade dairy alternatives prepared from almond, coconut and rice and bovine dairy milk showed significantly higher antiplatelet activity against PAF, in comparison to their non-fermented counterparts, with the rice-based drink displaying the strongest inhibitory activity.

Other sources of polar lipids include marine sources such as fish and algae [135]. Marine omega-3 PUFA are derived from fish, krill, and roe (fish eggs) and possesses significant antiplatelet activity [136], which may be more bioavailable in polar lipid forms. Polar lipid fractions isolated from codfish (*Gadus morhua*) showed platelet inhibitory capabilities, suggesting that consumption of such lipids could protect against cardiovascular disease [94]. Significant quantities of unused fish by-products by-catch and are generated from the fishing industry, including salmon heads, herring heads and off cuts, and boarfish. While these by-products and by-catch are conventionally regarded as undesirable, valorisation of their antithrombotic and cardioprotective properties could establish these products as important bioactive functional foods [137]. In a 2019 study, polar lipids derived from bycatch and by-products of these fish were assessed for their antiplatelet activity against various platelet agonists, and they exhibited strong inhibitory activities against PAF, thrombin, collagen, and ADP [89]. Another study focusing on salmon [90] demonstrated the potent *in vitro* antithrombotic effects of a food-grade polar lipid extract (FGE) prepared from salmon (*Salmo salar*) fillets in human platelets, in the presence of the platelet agonists PAF and thrombin. Among the lipid subfractions, phosphatidylcholines (PC) and phosphatidylethanolamines (PE) showed the strongest inhibitory capacity against PAF in human platelets. A later investigation found that salmon cooked *sous vide* at higher temperatures (80 °C and above) significantly reduced these antithrombotic properties, along with decreased PUFA content in salmon prepared without brining [138].

Another rich animal source of polar lipids is eggs. Egg yolks are a rich source of sphingomyelin, lysophosphatidylcholine (L-PC), and lyso-phosphatidylethanolamine (L-PE), along with other nutrients including protein, vitamins, and minerals [139,140]. Cage-free, organic, and daily fresh eggs were assessed to determine if their polar lipids exhibited antiplatelet properties. Out of the three varieties, lipid fractions from cage-free eggs showed the highest inhibition against PAF, owing mainly to the polar lipid component of the total lipid fraction [140]. Significant advances in poultry science have led to the natural fortification of eggs to contain higher levels of PUFA. It would be interesting to assess whether PUFA-rich eggs have different polar lipid compositions with even more effective antiplatelet properties considering the other potential cardioprotective effects that have been documented [141]. 

Overall, it appears that animal sources of polar lipids including dairy, meat, and egg products exhibit antithrombotic effects (Table 2). However, it should be noted that lipids sourced from non-animal sources such as vegetable oils are also known for their cardioprotective and antithrombotic properties, especially olive oil. A 2002 investigation [127] compared the *in vitro* antiplatelet properties of olive oil and other seed oils (sunflower, corn, sesame, and soybean) against PAF. Out of all the polar lipid samples, olive oil was the most bioactive and inhibited both PAF and thrombin in washed rabbit platelets [127]. Indeed, olive oil and related by-products have also been shown to affect PAF metabolism [142].

### 4.2. Ex Vivo and Human Studies

*Ex vivo* and human studies are important to conduct to gain an understanding of how polar lipids affect platelet and cardiometabolic homeostasis. Certain populations in which the local diet is rich in omega-3 PUFA, such as the Greenland Eskimos [145] and Mediterranean people [146] exhibit a lower rate of cardiovascular diseases. It has been speculated that dietary components such as polar lipids or PUFA may contribute to the observed benefits of these diets. As aforementioned, marine lipid sources, notably polar lipids and potentially PUFA sourced from oily fish species, exhibit antiplatelet activity. A 2019 crossover study involving healthy human volunteers found that intake of enriched marine oil supplements resulted in reduced platelet and leukocyte activation, among other beneficial effects on immune cell functioning [147]. However, similarly to the *in vitro* studies presented, foods and food derivatives other than marine sources exert antithrombotic effects. 

A recent investigation found that intake of yoghurt enriched with polar lipids from olive oil by-products resulted in lower platelet sensitivity against PAF and reduced low-grade inflammation, which was assessed by monitoring serum levels of IL-10 and IL-6 [97]. Alcoholic beverages are also known to contain anti-inflammatory and antithrombotic properties against PAF and other platelet agonists [148,149]. A crossover study found that the intake of Cabernet Sauvignon red wine and Robola white wine results in decreased postprandial platelet activity against PAF in human platelet-rich plasma (PRP) [150]. In this study, healthy male volunteers were provided with a standardized meal along with portions of either wine, ethanol solution or water, following which plasma samples were obtained at multiple time points. Platelet sensitivity against PAF was significantly affected following the intake of either red or white wine, compared to samples after intake of water in place of wines. Indeed, a related study investigated the consumption of wine and its effects on PAF metabolism and found that wine beneficially decreases the biosynthesis of PAF [151]. Collectively, these finding contribute to a growing body of literature that indicates there are bioactive constituents including polar lipids in alcoholic beverages such as wine [152] and beer [153]. Results from examples of these *ex vivo* studies are presented in Table 3. 

### 4.3. PAF Modulation by Micronutrients

Several dietary micronutrients such as vitamins, trace minerals and elements have exhibited anti-inflammatory, antithrombotic [154,155], and antioxidant functions [156] (Table 4). Among those, carotenoids, one of the main sources of vitamin A, are highly bioactive, with antioxidant, anti-inflammatory, and immunoregulatory properties [156,157]. The other form of vitamin A, retinol is known to affect PAF-R expression [158]. Vitamin E has also been linked to the metabolism of PAF and is capable of regulating platelet function [159]. A deficiency of vitamin E (alpha-tocopherol) was shown to stimulate the biosynthesis of PAF in rat polymorphonuclear leukocytes [160]. A study involving pregnant women found that oral supplementation with alpha-tocopherol inhibits platelet aggregation induced by ADP and PAF, using a range of concentrations from 6.55–500 mg/mL [161]. However, yet another *ex vivo* study in male volunteers found that short-term vitamin E supplementation does not significantly affect platelet function or phospholipase A_2_ (PLA_2_) and lyso-PAF activity [162], enzymes involved in PAF metabolism.

Vitamin D is a fat-soluble vitamin that exists in two major forms, namely cholecalciferol (D_3_) and ergocalciferol (D_2_). It is typically associated with bone and calcium homeostasis, and the risk of developing diseases such as osteoporosis and rickets [163]. However, vitamin D has diverse physiological functions and is involved in inflammatory and procoagulatory pathways in the body due to its important role in immune function [164]. A randomized study found that vitamin D supplementation can reduce platelet-mediated inflammation and oxidative stress in diabetic patients [165]. Vitamin D can also regulate haemostasis, and its deficiency is associated with increased platelet aggregation in the presence of the agonist ADP [166]. An *in vitro* experiment demonstrated that 25-hydroxyvitamin D, a metabolite of vitamin D, attenuated increased expression of *PTAFR* in a human respiratory epithelial cancer cell line in response to rhinovirus infection [167], indicating that vitamin D might regulate PAF-R expression. It has also been hypothesized that vitamin D may attenuate PAF signalling in other viral infections via the PAF-R such as in SARS-CoV-2 infection and coronavirus disease 2019 (COVID-19) [168]. Indeed, paricalcitol, a vitamin D analogue, is a known PAF-inhibitor as demonstrated *in vitro* and *in vivo* [169].

Vitamin C is a water-soluble vitamin abundantly found in plant sources such as citrus fruits and leafy vegetables. In addition to its well-documented roles in immune function and wound healing, vitamin C possesses antioxidant and antiplatelet functions [170]. In an *ex vivo* study, the addition of vitamin C effectively halted platelet aggregation and scavenged reactive oxygen species (ROS) in human platelets [171]. Another study found that dietary supplementation with vitamin C prevented the accumulation of PAF-LL agonists and cigarette-smoke-induced platelet adhesion and aggregation [172]. This also has important implications for vitamin C supplementation as a dietary intervention to reduce the risk of cardiovascular disease linked to smoking. These findings are in accordance with studies in rabbits that have shown that vitamin C downregulates PAF and PAF-LL and improves postischemic oxidative and inflammatory responses [173].

**Table 4:** Studies investigating the *in vitro*, *in vivo*, and *ex vivo* antiplatelet properties of micronutrients.

Micronutrient	Study Aim	Study Type	Result
Vitamin C	Effect of vitamin C on the release of PAF and PAF-like phospholipids during reperfusion injury.	*In vivo*	Vitamin C attenuated oxidative stress and reduced PAF and PAF-like lipid levels in rabbits [173].
Vitamin D	Study the effect of vitamin D supplementation in volunteers with Type 2 diabetes in a placebo-controlled trial.	*Ex vivo*	Six months of vitamin D supplementation decreased platelet activation and inflammatory markers such as IL-18, TNF-α and IFN-γ [165].
Vitamin D	Study the inhibitory effect of paricalcitol against PAF and thrombin-induced platelet aggregation	*In vitro*	Addition of paricalcitol effectively inhibited platelet aggregation as well as modulating the activity of metabolic enzymes PAF-CPT and PAF-AH in platelets and leukocytes [169].
Vitamin E	Establish the role of vitamin E (alpha-tocopherol) during pregnancy in platelet function	*In vivo*	Vitamin E supplementation almost completely inhibited platelet aggregation in presence of PAF and ADP, with very high inhibition observed in the brush border membrane vesicles [161].
Selenium (Se)	Investigate the mechanism by which selenium modulates PAF production in endothelial cells	*In vitro*	Selenium deficiency reduces PAF biosynthesis in bovine endothelial cells by downregulating the activity of anabolic enzymes [174].
Zinc (Zn)	Consequences of abnormal Zn storage and release in mouse platelets	*In vivo*	Ionic Zn^2+^ accumulated in secretory granules is released upon platelet activation and has a procoagulant effect [175].
Copper (Cu)	Role of dietary copper in platelet activation using rat models	*In vivo*	Platelet aggregation induced by ADP is significantly higher in copper-deficient rats compared to rats with an adequate amount of copper in their diet [176].

## 5. Importance of Essential Trace Metals on PAF-R Targets

Dietary trace metals are principal components and regulators of various metabolic processes in the body. These elements form only 5% of the average human diet and are typically required in doses of 1–100 mg daily in adults [177]. Trace elements such as zinc (Zn), and copper (Cu) have been shown to affect platelet function in health and disease, but these elements may also affect the PAF pathways. Deficiencies in the trace element Se have been shown to upregulate PAF production in human [178] and bovine endothelial cells [174], by enhancing the activity of two important enzymes involved in the remodelling pathway of PAF biosynthesis, PLA_2_ and lyso-PAF-AT.

Zinc (Zn^2+^) is a known antioxidant and anti-inflammatory agent [179]. In rat models, zinc deficiency studies have shown a decrease in platelet aggregation and impaired reactivity to agonists, including ADP and thrombin [94,137,138]. Furthermore, recent studies have shown that altered levels of zinc impact platelet reactivity in zinc deficient conditions [180]. Chelation of intracellular zinc can also inhibit the tyrosine phosphorylation cascade, which reduces platelet reactivity and aggregation *in vitro* [181]. In turn, increased dietary zinc increases platelet responses to ADP and thrombin in human plasma [180]. In line with this, zinc supplementation of 50 mg Zn/day demonstrated increased platelet reactivity and serum zinc levels in humans [182]. Zinc supplements have also been shown to decrease oxidative stress and the production of inflammatory cytokines in elderly individuals [179]. The role of zinc in platelet aggregation has, however, not been fully elucidated and some studies also suggest a direct inhibitory role of zinc. It has been suggested that zinc interacts with PAF at the functional receptor site or contiguous site due to its specific inhibition of PAF-induced platelet activation [183]. A further study has shown that zinc levels must be inversely proportional to PAF levels to carry out these inhibitory effects [184]. Additionally, zinc must be present before PAF exposure. This suggests that PAF and receptor binding may be limited by zinc and phospholipid (PAF) interaction [143,144]. This model is supported by zinc’s ability to bind to phospholipids in a 2:1–1:1 complex, particularly to the negatively charged phosphate groups [185]. 

Like zinc, copper is an essential trace metal for the human body. The delicate balance of copper levels in the body is crucial to maintaining terminal oxidation, elimination of free radicals, and iron metabolism [186]. Several studies have shown the effects of altered copper levels on platelet aggregation and thrombin activity. For example, a study using mice subjected to copper deficient diets demonstrated a significant increase in prothrombin time, a parameter used to evaluate blood clotting [187]. This was followed by another study in rats fed a copper-deficient diet (0.3 μg copper/g of diet), which demonstrated impaired platelet adhesion to endothelial cells with an increase in ADP-induced platelet aggregation [176]. However, an *ex vivo* study using blood samples obtained from males found that copper alone, as well as combined with manganese accelerated platelet activation and led to the deformation of erythrocytes [188]. Thus, balanced levels of copper are necessary for healthy platelet activation and aggregation. The relationship between PAF and copper has also been shown to be similar to that of iron in terms of oxidation of lipids and PAF-associated enzymes, whereby the iron-catalysed production of hydroxyl radicals can promptly and conclusively inactivate PAF acetyl hydrolase, which can lead to the prolonged inflammatory effect of PAF. Furthermore, metal-induced oxidative stress and superoxide can activate PAF acetyl hydrolase, increasing PAF levels [149,150]. Trace metals such as copper and iron may indirectly affect PAF signalling through increasing reactive oxygen species and lipid oxidation. 

The interplay between trace metals and the PAF/PAF-R pathway has clinical implications. For example, pre-eclampsia is one of many conditions characterized by increased platelet aggregation and superoxide production and has been linked to alterations in trace metal levels, such as a decrease in manganese, copper, and zinc. As such, precautions during pregnancy to ensure balanced levels of essential trace elements are necessary to avoid conditions such as pre-eclampsia [189,190,191,192,193,194]. Indeed, elevated magnesium (mg) appears to exert protective effects against lesion formation as well as antiarrhythmic and antihypertensive effects [195]. Collectively, these studies show the important of trace metals in PAF biology, but little is known about whether trace metals affect PAF-R expression or function. 

## 6. Conclusions and Future Perspectives

Although pharmaceutical options exist for PAF-R antagonists, they are sparse, and they are not currently utilized against CVD. However, targeting the inhibition of PAF via the PAF-R through dietary means may be a strategy to reduce the risk of atherosclerosis and CVD by reducing the activities of PAF. In this review, we have presented the *in vitro*, *in vivo*, and human studies that have examined the dietary inhibition of PAF. It appears that dietary PAF inhibitors exert their beneficial effects is through their anti-inflammatory and antithrombotic properties. Indeed, many authors have suggested that the longstanding beneficial effects of the Mediterranean diet may be due to the abundance of PAF inhibitors present in the diet. However, there is still a paucity of research investigating polar lipid consumption in humans. Although outside the scope of this review, there is also significant research in animals and humans demonstrating that polar lipids may be cardioprotective via modulating lipid metabolism. Collectively, these advances in research may lead to the development of dietary interventions or nutraceuticals with the aim to deliver dietary PAF inhibitors. However, there are vast gaps in our knowledge regarding the modulation of PAF-R expression directly in health and disease that requires further investigation.

## Figures and Tables

**Table 1:** Comparison of different studies investigating phytocompounds and their antithrombotic activities against PAF andother platelet agonists.

Phytocompounds	Scientific Name (Common name)	Optimum Dose Determined or Dosage Investigated	Study Outcomes
Polyphenols such as theaflavin and its gallolyl ester, geranyl gallate, farnesyl gallate and geranylgeranyl gallate.	*Camellia sinensis* (Tea)	Theaflavin and its galloyl esters (IC_50_ = 32–77 µM), geranyl gallate, farnesyl gallate and geranylgeranyl gallate (IC_50_ = 6.4–7.6 µM).	Tea polyphenol such as theaflavin and its other galloyl esters showed potential antithrombotic activity against PAF and inhibited an acetyltransferase involved in its biosynthesis [100].
Polar lipids	*Camellia sinensis* (Tea leaves)	TL (110 ± 50 µg/ µL), PL (34 ± 4 µg/µL) and NL (820 ± 460 µg/µL) from 30 min observation respectively.	Synergetic effect of the antithrombotic activity of tea polyphenol and PL were against PAF, thrombin, ADP, and collagen, due to their high unsaturated fatty acid content especially rich in omega-3 PUFA and MUFA [101].
Sulphonoglycolipid	*Polypodium decumanum* (Fern calaguala)	IC_50_ = 2 μM.	Sulphoquinovosyl diacylglycerol 1,2-di-*O*-palmitoyl-3-*O*-(6-sulpho-α-d-quinovopyranosyl)-glycerol showed inhibitory activity against PAF in an *in vitro* model using human neutrophils [102].
Curcumin	*Curcuma longa* (Turmeric)	Concentration: 0.3 mg/day in mice.	Oral administration of curcumin (0.3mg/day) in mice inhibited thromboxane levels and increased prostacyclin activity [103].
*Ar*-turmerone	*Curcuma longa* (Turmeric)	IC_50_ values of 14.4 µM and 43.6 µM against collagen and arachidonic acid (AA) respectively.	*In vitro* study showed that aromatic (ar-)turmerone effectively inhibits platelet aggregation induced by collagen and arachidonic acid [104].
Curcuminoids	*Curcuma longa* (Turmeric)	Concentration: 10–30 µg/mL.	The isolated PRP was exposed to various concentrations of curcuminoids (10–30 µg/mL) and showed antiplatelet activity against AA and collagen [105].
Allicin and thiosulfinates	*Allium sativum* (Garlic)	Volume: 30 μL of garlic juice.	*In vitro* studies showed that allicin and thiosulfanates are the key constituents of garlic juice resulting in antiplatelet activity against collagen-induced platelet activity [106].
Thiosulfinate	*Allium cepa* (Onion)	Volume: 220 µL of onion juice.	The study resulted that 220 μL of onion juice was enough to produce complete inhibition of platelet aggregation *in vitro* against AA [107].
AMP48 (Serine protease) of latex	*Artocarpus heterophyllus* (Jack fruit)	Amount: 1, 2, 4, 8, 16, 32 μg.	Using a thrombin and CaCl_2_ mediated fibrin clot experiment, 4 μg of AMP48 completely hydrolyzed α-subunit of fibrinogen in 30 min. Techniques including N-terminal sequencing fibrinolysis and ATR-FTIR spectroscopy revealed this novel protein has fibrinolytic properties [88].
Eugenol, amygdalactone, cinnamic alcohol, 2-hydroxycinnamaldehyde, 2-methoxycinnamaldehyde, coniferaldehyde, acetylsalicylic acid, coumarin, cinnamaldehyde, cinnamic acid, icariside DC, dihydrocinnacasside,	*Cinnamomum cassia* (Cinnamon bark)	IC_50_ values of Eugenol and coniferaldehyde obtained as 3.8 and 0.82 μM against AA; 3.51, and 0.44 μM against U46619 (thromboxane A_2_ mimic); 1.86 and 0.57 μM against epinephrine-induced aggregation.	Among the 13 compounds from the extract of cinnamon bark, eugenol, and coniferaldehyde were the two of the most active antiplatelet constituents against AA, U46619 (thromboxane A2 mimic) and epinephrine-induced platelet aggregation [108].
Aqueous extract from the bark	*Cinnamomum tamala* (Indian Bay Leaf)	Various concentrations of 100, 200, 300, 400, and 500 µg.	The aqueous extract inhibited TXB_2_ formation through COX pathway (IC_50_ of 112 µg ± 16) also LP-1 by LOX pathway (IC_50_ of 120 µg ± 15), and 500 µg concentration showed complete inhibition of platelet aggregation [109].
(6S,7Z,9R)-roseoside, Eriodectyol and 2″-O-rhamnosyl vitexin	*Crataegus pinnatifida* (Chinese hawberry)	Concentration: 400 µg/mL.	The isolated compounds 7, 13 and 15 exhibited potent antithrombotic activity against ADP induced platelet aggregation *in vitro* by 87.18, 72.92 and 75.00% respectively at 400 µg/ mL, among them the 13th compound exhibited antithrombotic activity *in vivo* (zebrafish) by prolonged thrombus formation (19.04 ± 3.32 min) than heparin control (17.63 ± 2.23 min) [110].
Ethanolic extract	*Ocimumbasilicum* (Basil)	Concentrations: 0.1, 1 and 10 mg/mL of Ocimum ethanolic extract.	Overall OBL and its extracts elevated 6-keto-PGF1α production while decreasing PGE_2_ and TXB_2_ production in a dose- and time-dependent manner. This might be due to the combined inhibition of COX-2 and activation of endothelial COX-1 [111].
Methanolic leaf extract	*Mangifera sylvatica* (Himalayan mango)	A volume of 100 µL.	Methanolic fraction showed a maximum of 46.93% clot lysis activity whereas streptokinase standard showed 80.51% [112].
Mangiferin	*Mangifera indica* L. (Mango)	Extracts from each part of the mango such as pulp, peel, seed husk and seed with various concentrations like 0.1, 0.5, and 1 mg/mL.	Mango seed showed a 72% of inhibition against adenosine 5′-diphosphate (ADP) induced by platelet aggregation. Among the identified monogalloyl compounds and benzophenones, mangiferin showed a 31% of inhibitory effect against ADP [113].
Bromelain	*Ananas comosus* (Pineapple)	Bromelain at various doses of 70, 140, and 210 μg/kg of body weight.	Antiplatelet aggregation tests from *in vivo* method exhibited that bromelain (at the dose of 210 μg/KgBW) has increased the bleeding time (515.10 ± 182.23%) on the 21st day of termination [114], indicating antiplatelet effects.
Baru almond oil	*Dipteryx alata* Vog (Baru Almond)	Ten days of Baru oil as 7.2 and 14.4 mL/kg/day.	Baru almond oil treatment has lowered about 31% of ADP-induced platelet aggregation and thrombotic processes in male Wistar rats, suggesting that it helps lower platelet activation and exert advantages in thrombotic processes [115].
Aqueous extract of strawberry fruit	*Fragaria ananassa* (Strawberry)	Extract concentrations from 0.1–1 mg/mL.	Dose-dependent reduction against AA and ADP-induced platelet aggregation was observed as 65 ± 5% and 55 ± 4% of inhibition respectively [116].
Hippuric acid	Phenol-rich fruits and plant	Concentrations: 100, 200, 500, 1 and 2 mM.	Dose-dependent inhibition against platelet surface receptor P_2_Y_1_/P_2_Y_12_ induced by ADP [117].
Piperine, pipernonaline, piperoctadecalidine, piperlongumine	*Piper longum* L. (Black Pepper)	Concentrations: 300, 150, and 30 μM.	The most effective antiplatelet agent was piperlongumine *in vitro*. Piperlongumine inhibited collagen-induced platelet aggregation with inhibition rates of 100, 100, 49.8, and 19.9% at 300, 150, 30, and 10 μM, respectively. Piperlongumine had 100%, 76.4%, and 12% inhibitory activity in an AA test at 300, 150, and 30 μM, respectively. Furthermore, piperlongumine at doses of 300, 150, and 30 μM reduced PAF-induced platelet aggregation with inhibition rates of 100%, 100%, and 29.9%, respectively [118].
Orientin and Iso-orientin	*Vaccinium bracteatum* Thunb. (Sea bilberry or Asiatic bilberry)	*In vitro* experiment with 5 to 50 μM and *in vivo* experiment with 9, 26.9 and 44.8 μg per mouse respectively.	A dose-dependent reduction in platelet aggregation was observed *in vitro*. *In vivo* experiments showed dose-dependent inhibition against thrombin was observed in mice model. From both compounds, orientin showed potent activity in both models [119].
Oleuropein	*Olea europaea* (Olive)	IC_50_ = 0.41 mM.	The various concentrations ranging from 0.25 to 1.25 mM of oleuropein has shown dose-based inhibition against PAF *in vitro* [120].
Gomisin N and pre-gomisin	*Schisandra chinensis* (Magnolia berry)	IC_50_ values of gomisin N and pre-gomisin as 96.5 and 153.3 μM against AA and 49.3 and 122.4 μM against PAF were obtained respectively.	From the various solvents extracts of *S. chinensis* fruit, methanol and hexane have shown higher inhibitory effects as 65.7 and 94.8% respectively against AA. When compared to all agonists such as PAF, AA, collagen and thrombin, compounds gomisin N and pre-gomisin showed higher effects against AA and PAF [121].
(+)- fenchone and estragole	*Foeniculum vulgare* Gaertner (Fennel fruit)	Concentrations: (+)- fenchone (IC_50_ values 3.9μM and 27.1 μM against collagen and AA) estragole (IC_50_ values 4.7 μM against collagen).	From the *in vitro* study, (+)-fenchone’s inhibitory effect against platelet aggregation caused by AA was 1.3 times greater than that of aspirin [122].
Pinocembrine, Alpinetin, Cardamonin, 2′,3′,4′,6′-Tetrahydroxychalcone, 5,6-Dehydrokawain, Flavokawain B (above all from *A. mutica*), Flavokawain A, Crotepoxide, 3-Deacetylcrotepoxide, Zerumbone (above all from *Z. zerumbet*), Xanthorrhizol (from *C. xanthorrhiza*), Curcumin, Xanthorrhizol epoxide, 1-Acetyl-2-methyl-5-(1′,5′-dimethylhex-4′enyl) benzene, 1-Methoxy-2-methyl-5-(1′,5′-dimethylhex-4′enyl) benzene (above all from *C. aromatica*)	*Alpinia mutica* Roxb. (Orchid Ginger) *Kaempferia rotunda* Linn (Blackhorm) *Curcuma xanthorhiza* Roxb (Javanese turmeric) *Curcuma aromatica* Valeton (Turmeric) *Zingiber zerumbet* Smith (Shampoo ginger)	Concentrations: 84 μM against AA and 45.7 μM against AA, collagen, and ADP.	Curcumin, cardamonin, pinocembrine, 5,6-dehydrokawain, and 3-deacetylcrotepoxide significantly inhibited platelet aggregation triggered by the AA with IC_50_ values less than 84 μM. Curcumin was the most efficient antiplatelet agent, inhibiting AA, collagen, and ADP-induced platelet aggregation with IC_50_ values of 37.5, 60.9, and 45.7 μM, respectively [123].
Vitamin C (Ascorbic acid) and total lipids (TL)	*Citrus sinensis* (Sweet orange) *Citrus sinensis* (Blood orange) *Citrus clementina* (Clementine)	IC_50_ values against PAF with various samples are as follows, Fresh juice of Navalina oranges (23.2 µg), sanguine oranges (21.4 µg), clementines (28.6 µg), TL from navalina (14.3 µg), TL from sanguine (15.3 µg), TL from clementines (17.3 µg), TL of navalina peel (1.5 µg), TL of sanguine peel (1.2 µg), TL of clementines (1.7 µg).	*In vitro* antiplatelet activity of vitamin C and TL extract of three different citrus fresh and oxidized fruit juice and peels have shown possible inhibitory effects against PAF and thrombin [124].
Aqueous extract of leaf	*Moringa oleifera* (Drumstick tree)	IC_50_ values against ADP-induced aggregation were 0. 48 mg and 0. 70 mg respectively.	Aqueous extract of moringa leaf (0.1 to 1mg) showed potent activity against all types of agonists used in this study such as collagen, ADP, and epinephrine. 1 mg of the extract has shown 100% inhibition against epinephrine-induced aggregation [125].
Ethanolic extract of grape pomace rich in phenolics (catechin, epicatechin and quercetin) fatty acids (linoleic acid (C18:2n6), linolenic acid (C18:3n3) and palmitic acid (C16:0))	*Vitis vinifera* (Grape tree)	IC_50_ value against PAF, ADP, and TRAP as 160.7 ± 64.2, 180.8 ± 78.8, and 158.1 ± 93.6 μg, respectively.	From the *in vitro* antiplatelet activity, the ethanolic extract of grape pomace was found to be rich in phenolics and fatty acids such as linoleic, linolenic, and palmitic acid. The IC_50_ values were calculated as 144, 176.5 and 180.5 μg of extract (healthy volunteer) and 214.2, 191.8 and 177.1 μg of extract (cardiovascular patient) against PAF, ADP and TRAP respectively [126].
Olive oil rich in glycerol−glycolipid	*Olea europaea* (Olive)	IC_50_ values of Polar lipid fractions 3 showed 437.5 μL, 4 showed 162.5 μL and 5 showed 375.0 μL against PAF.	From the various olive oil fractions, it was evident that glycerol-glucolipids, phosphatidylcholine, sphingomyelin, phosphatidylinositol, and phosphatidylserine were identified and have potent antiplatelet activity against PAF [127].

**Abbreviations:** ADP, adenosine diphosphate; AA, Arachidonic acid; COX_2_, Cyclooxygenase-2; HUVEC, human umbilical vein endothelial cells; IC_50_, 50% inhibitory concentration; 6-keto-PGF1α, 6-keto prostaglandin F1α; MUFA, Monounsaturated fatty acids; NL, neutral lipids; OBL, *Ocimum basilicum* L; PAF, Platelet activating factor; PGE_2_- Prostaglandin E_2_; PL, polar lipids; PUFA, Polyunsaturated fatty acids; TL, Total lipids; TXB_2_, Thromboxane B_2_; TRAP, Thrombin receptor activator peptide.

**Table 2:** Comparison of in vitro studies investigating dairy and marine lipids possessing antithrombotic activity against PAF and other platelet agonists.

Lipid Source	Study Aim	Result
Fermented Irish ovine yoghurt milk	Comparison of *in vitro* inhibition against PAF-induced aggregation, among different yoghurts and unfermented ovine milk.	Fermentation enhances the antiplatelet nature of ovine milk, due to specific starter cultures, e.g., *Lactobacillus* (demonstrated by decreased IC_50_ values) [132].
Fermented bovine yoghurts and coconut, almond and rice-based dairy alternative drinks	Comparison of *in vitro* inhibition by PL of platelet aggregation.	Fermented plant-based dairy alternatives show much higher antiplatelet activity compared to non-fermented counterparts. The PL from rice-based fermented products shows the highest platelet inhibition of all products, against aggregation induced by PAF and ADP [134].
Kefalotyri and Ladotyri Greek cheeses	Investigate the *in vitro* inhibition of cheese PL against PAF-induced aggregation.	Lipid fractions of both kinds of cheese inhibit platelet activation, Ladotyri has stronger inhibition [96].
Greek yogurts derived from cow, ewe, and goat milk	Evaluate the *in vitro* anti-thrombotic properties of yogurts in presence of PAF.	TPL and TL of all yogurts showed platelet inhibition, with TPL of goat and ewe yogurt demonstrated highest inhibition against PAF in WRP [143].
Irish organic farmed salmon filet	Investigate the *in vitro* inhibition by salmon PL extract against PAF and thrombin-induced platelet aggregation.	Salmon PL, TNL and TL fractions from PE and PC showed higher inhibitory activity [90].
Fresh and fried cod (*Gadus morhua*)	Test the PAF-like and anti-PAF properties of lipid fractions of fresh and fried cod, against PAF-induced platelet aggregation.	Lipid fractions (TPL and TNL) from fried and fresh cod showed inhibitory activity as well as slight platelet aggregation, indicating presence of both PAF agonists and inhibitors [94].
Hen’s egg yolk	Comparison of the antiplatelet activity of TL, TPL and TNL of different types of hen’s egg yolk (daily fresh, organic, and cage-free hen’s eggs).	All 3 types of hen’s egg yolks displayed potent inhibition against PAF-induced aggregation, with cage-free egg yolk having the highest bioactivity of all, in washed rabbit platelets (WRP) [140].
Red and white wines and musts	Assess the biological activity of lipid fraction from wines/must *in vitro*.	All lipid fractions of all samples exhibited inhibition against PAF-induced aggregation in washed rabbit platelets, with TPL of Ambelon (white wine) and Cabernet Sauvignon (red wine) having the most potent antiplatelet activity of all [144].

**Abbreviations:** ADP, adenosine diphosphate; TNL, total neutral lipids; PAF, platelet-activating factor; PC, phosphatidylcholine; PE, phosphatidylethanolamine; PL, polar lipids; TL, total lipids; TPL, total polar lipids; WRP, washed rabbit platelets.

**Table 3:** Studies investigating the *ex vivo* antiplatelet properties of animal lipids and alcoholic beverages.

Lipid Source	Study Aim	Study Type	Number of Volunteers	Control	Result
Marine oil omega-3 supplement	Establish the relationship between marine oil supplementation and specialized pro-resolving mediators (SPM)	A double-blinded, placebo-controlled crossover	22	Placebo	Platelet aggregates induced by PAF stimulation are reduced after consumption of marine oil supplement [147].
Yoghurt enriched with olive oil pomace polar lipids	To determine the effect of the incorporation of olive oil pomace polar lipids in yoghurt and their effects on platelet function	Randomised double-blinded, placebo-controlled	30	Plain yoghurt	Consumption of yoghurt enriched with olive oil PL resulted in lower platelet sensitivity to PAF [97].
Cabernet sauvignon red wine or Robola white wine	Assess the beneficial effects of wine intake in the postprandial state in human volunteers	Crossover study	10	Water and ethanol	Consumption of red or white wine along with a standardized meal resulted in reduced postprandial PAF-induced platelet aggregation in healthy male volunteers [150].
